# Divergence between functional magnetic resonance imaging and clinical indicators of language dominance in preoperative language mapping

**DOI:** 10.1002/hbm.25092

**Published:** 2020-06-10

**Authors:** Antonina Omisade, Christopher O'Grady, R. Mark Sadler

**Affiliations:** ^1^ Acquired Brain Injury (Epilepsy Program), Nova Scotia Health Authority Halifax Canada; ^2^ Department of Psychology & Neuroscience Dalhousie University Halifax Canada; ^3^ Department of Research Nova Scotia Health Authority Halifax Canada; ^4^ Department of Medicine, Division of Neurology Dalhousie University Halifax Canada; ^5^ Biomedical Translational Imaging Centre Halifax Canada

**Keywords:** epilepsy surgery, functional MRI, language laterality

## Abstract

Accurate determination of hemispheric language dominance prior to epilepsy surgery is critically important to minimize cognitive morbidity. Functional MRI (fMRI) is a noninvasive method that is highly concordant with other clinical indicators of language laterality, and is now commonly used to confirm language dominance. However, there is also a high frequency of divergence between fMRI findings and other clinical indices that complicate determination of dominance and surgical decision‐making in individual patients. Despite this, divergent cases are rarely published or discussed. This article provides three illustrative examples to demonstrate common scenarios where fMRI may produce conflicting or otherwise difficult‐to‐interpret findings. We will also discuss potential reasons for divergence and propose a flow‐chart to aid clinical decision making in such situations.

## Graphical abstract

There is a high frequency of divergence between functional MRI (fMRI) findings and other indices of hemispheric language dominance that complicate clinical decision‐making in individual patients prior to neurosurgery. Despite this, divergent cases are rarely published or discussed. This article provides three illustrative examples to demonstrate common scenarios where fMRI may produce conflicting or otherwise difficult‐to‐interpret findings, and includes a flow‐chart to aid clinical decision making in such situations.

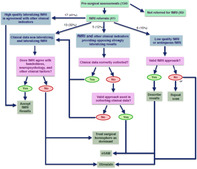

## 1 INTRODUCTION

People with medically refractory focal onset epilepsy may be candidates for surgical resection of epileptogenic regions to help control their seizures. Epilepsy surgery has been shown to be a safe and effective procedure, with rates of postoperative seizure freedom at 50–75% depending on the location of the lesion and contributing pathology. By contrast, the chances of achieving seizure freedom after unsuccessful trials of two drugs (provided good medication compliance and no significant adverse side effects) is <5% and continues to decline with each unsuccessful drug trial (Elger & Schmidt, 2008; Kwan & Brodie, 2000). Early epilepsy surgery has been shown to reduce the risk of morbidity and improve quality of life over the long term compared to drug treatment alone (Engel et al., 2003; Engel et al., 2012). However, surgical resections carry certain risks that are dependent on the location and extent of surgical resection. Although risk of adverse effects like stroke or paralysis are small, (around 1–2% [Kaufmann et al., 2007]), the risk of postoperative language and memory morbidity, when surgery involves the temporal lobe, are significantly greater. These changes are more likely to be particularly severe or functionally‐significant if the surgery affects the language‐dominant hemisphere (e.g., Sherman et al., 2011). Accurate determination of hemispheric language dominance prior to epilepsy surgery is an essential component of preoperative planning, with functional MRI (fMRI) emerging as the leading noninvasive method for language lateralization (Bauer, Reitsma, Houweling, Ferrier, & Ramsey, 2014; Binder, 2011).

The concordance between fMRI and other clinical methods of determining language dominance, including seizure semiology, EEG, and structural imaging findings, neuropsychological tests, MEG, and invasive intracarotid deactivation procedures (like Wada), has been reported to be up to 90% (Binder, 2011; Gargaro et al., 2013; Woermann et al., 2003). However, high agreement among these modalities is restricted to a very specific and highly selected group of patients, namely individuals with strong left hemisphere language dominance and temporal (vs. extra‐temporal) seizure foci (some representative articles include: Bauer et al., 2014; Hund‐Georgiadis, Lex, Friederici, & von Cramon, 2002; McDonald, 2008). In individuals with right hemisphere language or some form of atypical dominance pattern, such as equal language representation in both hemispheres (bilateral dominance) or expressive and receptive language functions in different hemispheres (crossed dominance), concordance rates among these methods is as low as 50–60% (Bauer et al., 2014). In a recent survey completed by Benjamin et al. (2018), 54% of surgical programs in the United States, Canada, Australia, and Europe reported instances of disagreement between fMRI results and other methods noted earlier. At the same time, Benjamin et al. (2018) reported that out “of all programs reporting disagreement between fMRI and another measure,… 5% had published these cases.” It is also unclear how the discrepancy was ultimately resolved (if at all) with respect to clinical decision‐making. As such, circumstances leading to disagreement among methods and its impact on clinical decision‐making are under‐explored and poorly understood.

This article describes three illustrative cases, which were selected from among all presurgical evaluations completed since fMRI has been in routine clinical use in our center since 2015. These cases were selected to demonstrate three main types of fMRI discordance with other methods that we have encountered in our surgical epilepsy program. We also discuss some possible reasons for discordance and propose a clinical decision‐making algorithm to increase the accuracy of these determinations.

## 2 METHODS

At the QE II Health Sciences Centre (HSC) in Halifax, Nova Scotia, all patients undergoing standard presurgical work‐up for epilepsy receive at least the following: inpatient video EEG telemetry monitoring, 3 Tesla structural MRI, and neuropsychological testing. The tests included in the presurgical neuropsychological assessment battery are listed in Table 1. Language dominance is inferred based on the neuropsychological profile and its convergence with other clinical findings. It is confirmed using the dichotic listening test (Kimura, 1967).

**TABLE 1:** Results of presurgical neuropsychological testing for three patients

	Case 1		Case 2		Case 3	
Test	Score	Rating	Score	Rating	Score	Rating
WASI‐II (standard scores)						
FSIQ	99	Average	73	Borderline	84	Low average
VCI	101	Average	80	Low average	77	Borderline
Vocabulary	50%ile	Average	10%ile	Low average	7%ile	Borderline
Similarities	55%ile	Average	14%ile	Low average	9%ile	Low average
PRI	97	Average	70	Impaired	95	Average
Matrix reasoning	50	Average	2%ile	Impaired	22	Low average
Block design	39	Average	6%ile	Borderline	58	Average
BNT (*T* score)	40	Low average	42	Low average	29	Impaired
Verbal fluency (*T* scores)						
Letter	44	Average	42	Low average	37	Low average
Semantic	39	Low average	44	Average	30	Borderline
Jones–Gotman design fluency (*z* scores)						
Free condition	2.38	Superior	−1.72	Borderline	−1.56	Borderline
Fixed condition	−0.34	Average	−2.84	Impaired	−1.59	Borderline
Complex figure (*z* scores)						
Copy	−1.43	Borderline	−0.46	Average	0.26	Average
Recall (30 min)	−1.20	Low average	−0.13	Average	−1.35	Borderline
WMS‐III (standard score)						
Logical memory I	11	Average	6	Low average	7	Low average
Logical memory II	9	Average	9	Average	7	Low average
RAVLT (raw/*z* scores)						
Trial 1	8/0.82	High average	6/−0.13	Average	3/−2.12	Borderline
Trial 5	14/0.89	High average	13/0.43	Average	9/−1.74	Borderline
Long delay (20 min)	10/−.07	Average	13/0.97	High average	5/−1.86	Borderline
AFLT (raw scores)						
Trial 1	2	Normal	7	Normal	3	Normal
Trial 5	3	Low	12	Normal	7	Low
Long delay (20 min)	3	Normal re: Trial 5	11	Normal re: Trial 5	6	Normal re: Trial 5
Munn faces (raw score)	8	Borderline	9	Normal	7	Impaired
SDMT—Written (*z* score)	−1.09	Low average	−0.03	Average	−0.39	Average
D‐KEFS (standard scores)						
Color‐word	11	Average	8	Average	—	—
Trails	8	Average	5	Borderline	—	—
WCST						
Categories	6	Average	—	—	6	Average
Perseverative errors	6%	Average	—	—	10	Average
Set loss	0	Average	—	—	1	Average
FDWT ear difference score (R − L)	−7	NEA with slight left ear preference	3	NEA	−12	LEA

*Note*: AFLT, Aggie figural fluency test; BNT, Boston naming test; Color‐word: only Condition 3 (interference) is reported; Complex figure, Rey–Osterreith complex figure test; Design fluency, Jones–Gotman design fluency (total score for free and fixed conditions reported); D‐KEFS, Delis–Kaplan executive function scales; FDWT, fused Dichotic words test; RAVLT, Rey auditory verbal learning test (Trials 1 and 5 are immediate recall trials, long delay is free recall of after 20 min); VCI, verbal comprehension; Verbal fluency‐letter, Thurstone verbal fluency (letters D and C), Verbal fluency—semantic, Animals; WASI‐II, wechsler abbreviated scale of intelligence‐II; WMS‐III, Wechsler memory scales‐III; SDMT, symbol‐digit modalities test; Trails: only condition 4 (switching) is reported; WCST, Wisconsin card sorting test.

Dichotic listening involves presentation of similar but different verbal material (usually words) to right and left ears simultaneously over a number of trials. Since contralateral cochlear projections are predominant (Hugdahl, 2000), information that enters the left ear is first processed predominantly in the right hemisphere before being projected via the corpus callosum to the left hemisphere, and vice versa. Greater accuracy in reporting words delivered to one of the ears is associated with language dominance (right ear preference indicates left hemisphere dominance). The dichotic paradigm used in preoperative testing at our center is the Fused Dichotic Words Test (FDWT; Wexler & Halwes, 1983). A difference of 10 words between ears is considered to be the minimum cut‐off for a reliable ear advantage that may be indicative of hemispheric language dominance (Zatorre, 1989).

At our center, patients may be referred for fMRI following neuropsychological testing if there is an increased probability of atypical language representation. This decision may be based on several factors including: (a) a lack of reliable ear advantage (NEA) on dichotic listening, (b) left‐handedness or ambidexterity, and (c) neuropsychological profile is inconsistent with side of seizure onset (e.g., impaired visual memory in a patient with left temporal lobe epilepsy). From April 2015 until April 2019, 134 patients were seen for presurgical assessment for epilepsy, and 41 patients were referred for/completed fMRI for the reasons outlined above. The combination of neuropsychological tests and other lateralizing tests or factors such as dichotic listening, handedness, etc. with fMRI is used widely as part of presurgical planning. One study reported 91% of epilepsy surgery centres employing such a combined approach with fMRI paradigms most commonly used included word generation, naming, semantic comparison, listening, and reading tasks (Vogt et al., 2017). In another work, 96% of programs used fMRI and 99% use neuropsychological assessment as part of presurgical consideration (Benjamin et al., 2018).

For the cases described below, functional images were acquired on a 3 Tesla GE MR750 scanner using an echo‐planar pulse sequence with TR/TE = 2000/25 msec, FOV = 22 cm, FA = 77°, 48 axial slices (3 mm), in‐plane voxel resolution =1.72 mm. fMRI data processing and analyses were performed using AFNI (February 2015; Cox, 1996). Data was processed using the general linear model (Friston et al., 1994). The following processing steps were applied: standard rigid‐body realignment to the first volume, slice timing correction, spatial smoothing using Gaussian kernel of FWHM 4.0 mm, and removal of low–frequency signal drift. Processed functional images were registered to a T1 FSPGR image with TR/TE = 8.3/3.3 msec, FOV = 22 cm, FA = 12°, and 80 axial slices (1 mm), and in‐plane voxel resolution = 0.86 mm for anatomical localization.

**FIGURE 1:**
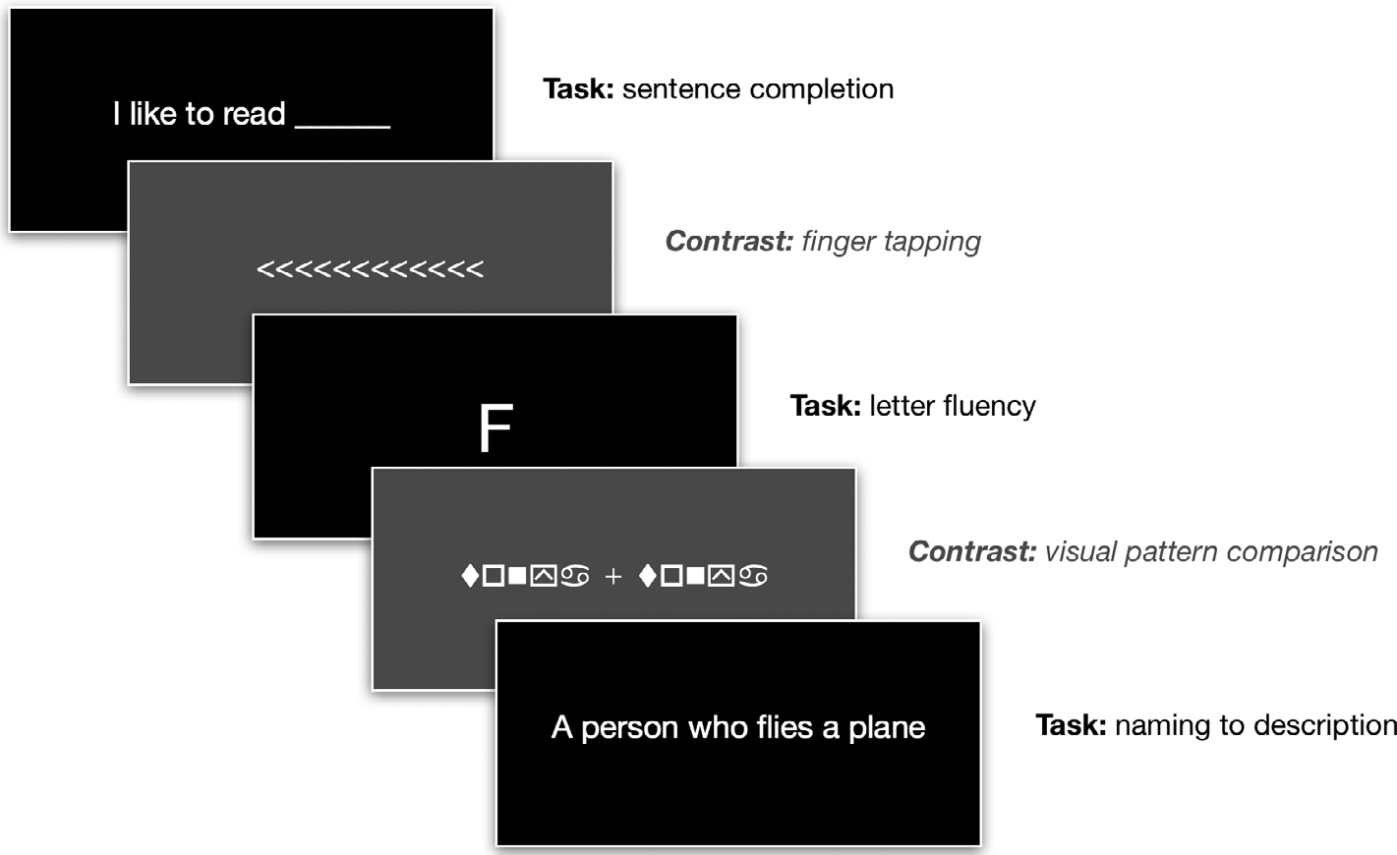
Diagram of the language fMRI paradigm (contrast conditions are in gray)

**FIGURE 2:**
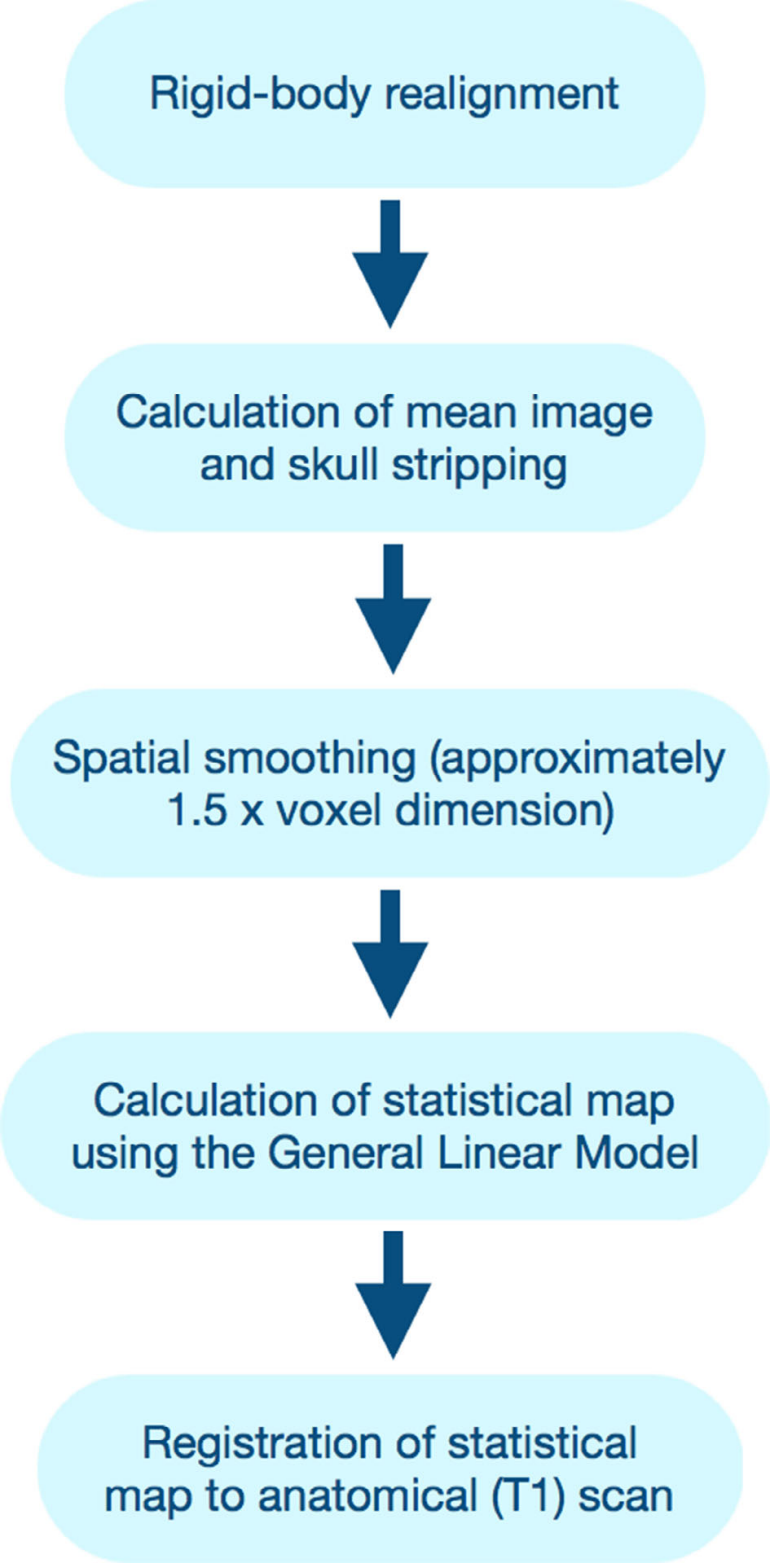
Flow chart of the fMRI processing pipeline

The language paradigm (Figure 1) is based on a clinical paradigm employed at the Toronto Western Hospital (M. P. McAndrews, personal communication, July 6–7, 2015) and consists of three language tasks presented visually and involve covert verbal responses: sentence completion (e.g., “I like to read ______”), letter fluency, and naming to description (e.g., “a person who flies a plane”—“pilot”). Each stimulus was presented for 3 s for sentence completion and naming to description. For letter fluency, each letter was presented for 12 s. The paradigm was block design with task blocks lasting 24 s, contrast blocks of 24 s, and 1‐s visual warnings for both the task and contrast blocks. The control tasks consisted of visual pattern comparisons and finger tapping, and were interleaved with the language tasks in a single run lasting 7:18 min. Some individuals with greater cognitive difficulties, particularly slower processing speed or language dysfunction, completed the same paradigm with naming and sentence completion stimuli presented at half speed (6 s per item). The visual control pattern task was used to activate the visual cortex to remove occipital activation. Finger tapping was included as a contrast to counteract any extraneous motor and sensory activation because some studies have shown even covert language tasks can elicit nonlanguage‐related activation in various frontal and posterior cortical regions (Desai, Binder, Conant, & Seidenberg, 2009; Glenberg et al., 2008). Postprocessing steps for fMRI data are detailed in Figure 2.

Thresholds for the statistical language maps produced using the GLM were chosen as the top 97th, 98th, or 99th percentile of *t*‐statistics (Gross & Binder, 2014), depending on the neuropsychologist's discretion. The clearest representation of language dominance was taken as that which minimized activation in non‐language areas, spurious activation, and other activation clearly not related to the language tasks.

**FIGURE 3:**
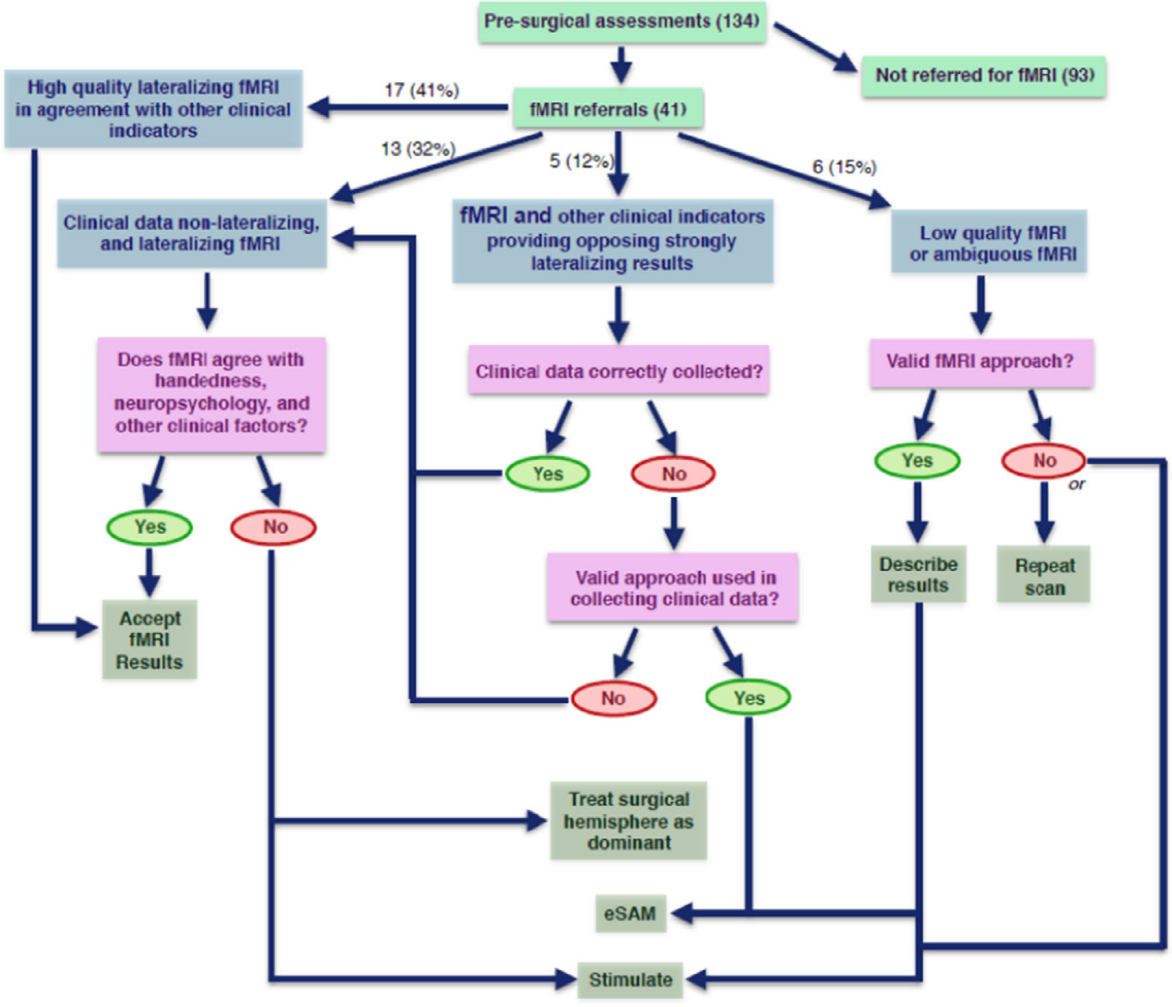
Flow chart to guide clinical decision making when using fMRI and dichotic listening for language lateralization

We have documented three types of common scenarios observed in our center where fMRI was difficult to interpret within the context of the overall clinical profile. These scenarios include: (a) Strongly lateralized fMRI, but ambiguous cognitive findings, (b) Low quality or ambiguous fMRI maps, (c) fMRI and other clinical indicators providing opposing strongly lateralizing results. Out of the 41 patients scanned at our center, 24 (59%) have fallen into one of the three categories. We discuss each scenario using illustrative cases. We include a decision tree that illustrates how the issues of discordance were resolved (Figure 3).

### 2.1 Strongly lateralized fMRI, but ambiguous cognitive findings

A presurgical evaluation in epilepsy focuses on examining discrepancies between verbal and visual/nonverbal abilities across cognitive domains to lateralize language and areas of dysfunction (presumably related to seizure focus). Verbal abilities are used as a marker for the functioning of the dominant hemisphere, while visuoperceptual/nonverbal abilities are used as a marker for the functioning of the nondominant hemisphere. Along with dichotic listening and seizure characteristics, this discrepancy can help determine the side of language dominance as well.

In some cases, patients do not show a clear and consistent discrepancy between verbal and nonverbal abilities on neuropsychological testing despite a clearly‐lateralized seizure focus, or do not obtain a clear ear advantage on dichotic listening. However, their fMRI maps produce very strongly lateralized findings. In this situation, we have weighed the decision more heavily toward fMRI findings with respect to determining language dominance, especially when the side indicated by fMRI was concordant with other surrogate markers of language dominance like handedness and side of seizure onset. In cases where other markers were also ambiguous, we have recommended treating the surgical hemisphere as dominant and avoiding traditional language zones or doing direct cortical stimulation in case resection would affect language zones. This was also the recommendation made in rare situations where fMRI maps also showed bi‐lateral activation. Thirteen of the 41 scans (32%) fell into this category. Not all of these patients would require an eSAM, however. Only in situations where language dominance was relevant in predicting risk of postoperative amnesia, invasive intracarotid deactivation testing (Wada or etomidate Speech and Memory Test (eSAM; Jones‐Gotman et al., 2005) was recommended.

*Case example 1*: 40‐year‐old, right‐handed woman with a college education, who has a 20‐year history of focal onset epilepsy. Her typical seizures begin with a feeling of terror, which may be followed by loss of awareness and arrest of behavior. Her speech was reportedly undisturbed at the start of the seizure, though it is unclear how often she has attempted to communicate during the very brief time between seizure onset and loss of contact. MRI showed findings in keeping with right hippocampal sclerosis. Telemetry EEG recordings demonstrated right temporal interictal abnormalities and right temporal onset seizures.

The results of the presurgical neuropsychological testing are detailed in Table 1. Her overall profile showed IQ and cognitive functions in most domains within the average range, which is consistent with her level of academic achievement. Nonverbal learning and visual memory represented clear areas of relative weakness in the profile. Although her performance on tests of verbal learning and memory were generally within normal limits, there was a greater‐than‐expected decline in recall on a word list after a 30‐min delay period, which is not consistent with the rest of the profile. The dichotic listening task demonstrated a slight left ear preference that did not quite reach threshold for reliability (i.e., 10‐point ear difference). An fMRI to confirm language dominance was recommended.

**FIGURE 4:**
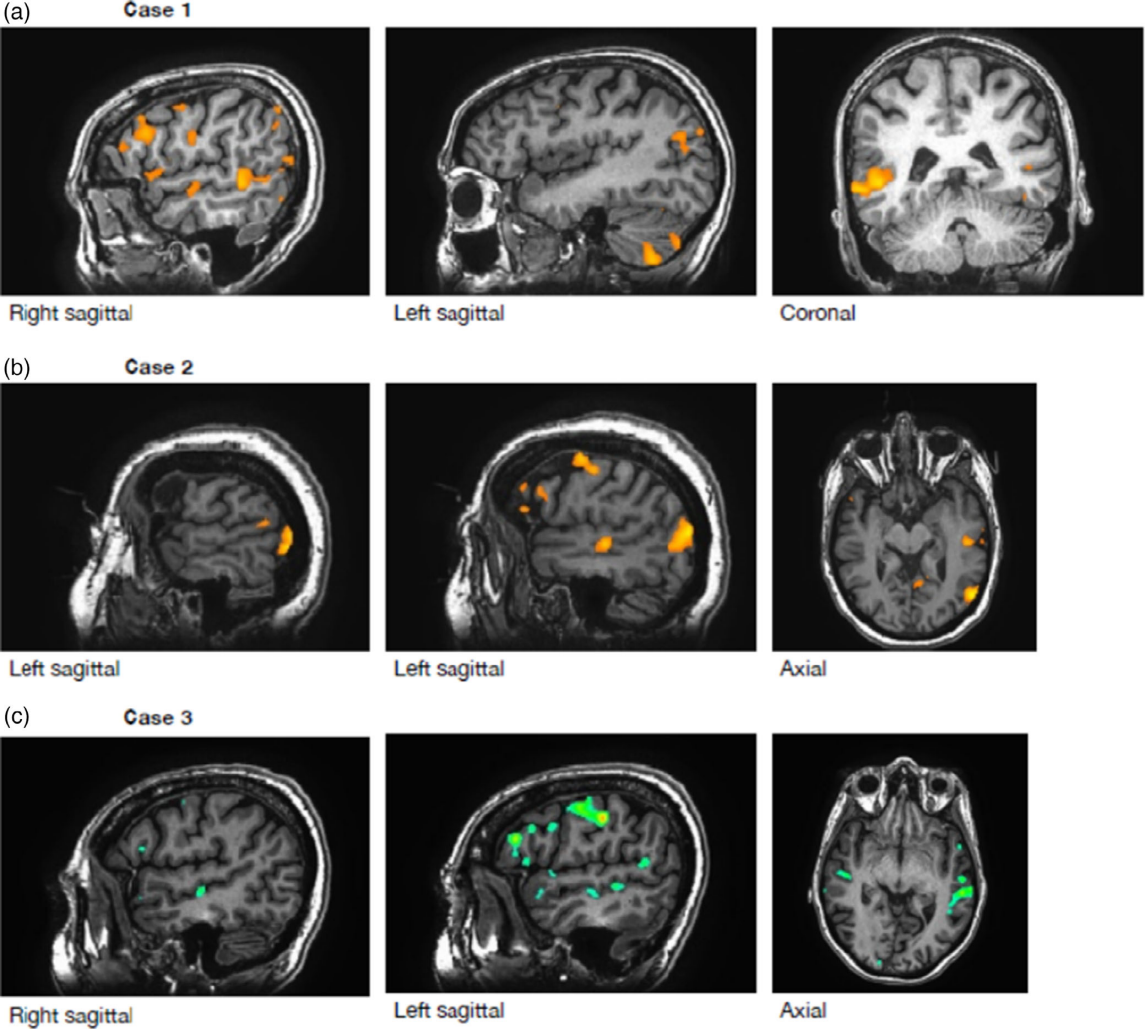
Selected images of the fMRI language maps for the three cases presented

fMRI language maps for Case 1 are presented in Figure 4a. Language‐related activation was noted predominantly in the *right hemisphere*, in regions traditionally associated with the language network: anterior and posterior temporal lobe, superior temporal gyrus, and inferior frontal lobe (Broca's area). In the *left hemisphere*, language‐related activation was observed in the cerebellum, which supports *right hemisphere dominance* (Stoodley, Valera, & Schmahmann, 2012). Robust, but very small areas of activation were also noted along the superior temporal gyrus, which suggests some language representation on the left, but was insufficient to conclude left dominance.

Right language dominance is highly unusual for right‐handed individuals, with only about 5% prevalence in the general population (Isaaks, Barr, Nelson, & Devinsky, 2006). Some factors that have been associated with atypical language representation and discordant findings among language dominance markers include increased age, nonfamilial left‐handedness and having no dominant hand for writing (Hund‐Georgiadis et al., 2002). None of these factors were applicable in this case. However, the influence of extraneous variables on dichotic listening performance, such as preferential attention to one ear (e.g., Hiscock & Kinsbourne, 2011; Hugdahl et al., 2003), could not be ruled out. By contrast, it is highly unlikely to obtain very strongly‐activating fMRI findings in traditional language zones on the side that is not dominant for language. Any technical factors that might affect the validity of fMRI would more likely create low quality or uninterpretable maps than clearly lateralized activation in the traditional language zones.

The neuropsychologist recommended that the right hemisphere be treated as dominant for the purposes of a right temporal resection. Although, assuming right hemisphere dominance increases this patient's risk of a significant decline in memory abilities after surgery, she was not considered to be at risk for anterograde amnesia and eSAM was not deemed necessary. To date, the patient has elected to continue medical treatment and there are no plans for surgery at this time.

### 2.2 Low quality or otherwise ambiguous fMRI

This refers to situations where fMRI maps were ambiguous due to insufficient activation within language regions, evenly bi‐lateral activation, excessive spurious activation, or language activation noted at very low thresholds (note: what is considered a low vs. high threshold for activation would vary between scanners and on language and control tasks selected. In this instance, we typically see language activation at *t* > 5, so anything below *t* = 4 would be considered low). Fortunately, the scenario of low *t*‐statistic values has been exceptionally rare in our center. The only common factor shared by most individuals in this category has been low IQ on neuropsychological testing and generally below‐average cognitive profile, which re‐enforces the guideline of avoiding fMRI with IQ scores below 70 (Binder, 2011). In these situations, it is especially important to ensure that the patient understood the instructions and was able to perform the tasks correctly in the scanner. This can be, in part, determined during the prescan practice session, which is critical for all individuals undergoing this procedure. Since in many centers, including ours, performance is not directly monitored in the scanner, a postscan debriefing session is important to get the patient's perspective on their performance (Benjamin et al., 2018). In fact, during one such interview, we determined that the patient had their eyes closed in the scanner as a result of misunderstanding instructions, necessitating a repeat of the procedure.

If it is determined that the tasks were understood and performed correctly, the results of the scan were described in detail in the report with a recommendation to treat the surgical hemisphere as dominant. eSAM would be recommended if there is a risk of catastrophic memory impairment following operation on the “dominant” side. This is in concordance with recommendations of confirming language dominance using Wada (or alternatively, eSAM) when fMRI indicates language dominance ipsilateral to the surgical hemisphere (Barr & Morrison, 2015). Fortunately, this scenario of a low quality or otherwise invalid fMRI scan is relatively rare with only 6 out of the 41 scans (15%) falling into this category.

*Case example 2*: 53‐year‐old, ambidextrous woman with a Grade 7 education, which involves 8 years of formal education (including grade Primary or Kindergarten) and is typically associated with Developmental Reading Assessment (DRA) level of 70 (Beaver, 2003). She presented with a history of focal epilepsy since childhood. Her seizures begin with a sense of “wooziness” followed by loss of awareness, lip smacking, a head turn to the left. She reported problems with speech and verbal comprehension for about 15 min following her seizures. Scalp video EEG telemetry recordings demonstrated right anterior temporal spikes and seizures. MRI was normal.

This patient's neuropsychology testing results are shown in Table 1. The profile was generally in the borderline to low average range, in keeping with her academic background. Impaired performance was noted on tasks of visuospatial reasoning, visuospatial construction, and nonverbal fluency, which was interpreted to be suggestive of nondominant hemisphere dysfunction, mostly affecting the frontal and, to a lesser degree, parietal regions. Her dichotic listening score did not show a reliable ear advantage. As a result of this, as well as her ambidexterity, an fMRI was recommended prior to surgical intervention.

Language maps for Case 2 are presented in Figure 4b. The paradigm primarily activated areas outside of the “traditional” language zones in regions that are often considered secondary in performance of the language tasks used in this study (e.g., occipital lobe and motor strip). This may be due to the patient becoming periodically distracted or otherwise having difficulties performing the language tasks, despite her self‐report to the contrary. Within the regions associated with the core language network, significant regions of activations were noted in the left hemisphere only. Specifically, small but reliable activation was noted in the posterior region of the superior temporal gyrus, and in the superior temporal sulcus. Surprisingly, there was no clear activation in the region of Broca's area, though other nearby regions within the prefrontal cortex were active.

The scan was considered to be of poor quality and showed many areas of robust activation in regions outside of the core language network. However, task‐related activation was also noted within the expected regions in the posterior left temporal lobe and in the left superior temporal sulcus; there was no reliable language activation on the right. Based on these findings, the probability of left hemisphere language dominance was considered high. Since this patient performed very well on both verbal and nonverbal memory tasks, she would likely experience a decline in her memory functions postoperatively, but would not be at risk for anterograde amnesia or catastrophic memory loss regardless of side of resection, so eSAM was not recommended. However, given the poor quality of the scan, it was considered advisable to either avoid interfering with regions corresponding to Broca's and Wernicke's areas on the right during surgery, or to stimulate areas around the resection site if it were to infringe on these traditional language zones. This patient was scheduled for a right temporal lobe resection, but was lost to follow up; surgery is not currently planned.

### 2.3 fMRI and other clinical indicators provide opposing, but strongly lateralizing results

In situations where clinical markers for language dominance and fMRI maps are clearly and strongly lateralizing, but in opposite directions (e.g., a strong LEA on dichotic listening combined with left language activation on fMRI), it is important to determine the patient's approach to cognitive testing that might produce falsely lateralizing findings or, in case of discrepant dichotic listening results, explore potential unilateral hearing loss. If other markers are deemed valid, we have avoided making a decision regarding laterality in these patients based on fMRI, even within the context of other clinical information. This is the group that is most likely to require an eSAM test to determine hemispheric language laterality. To date, 5 out of the 41 scans (12%) fell into this category.

*Case example 3*: 40‐year‐old, ambidextrous woman with a Grade 10 education and a 4‐year history of medically intractable focal seizures. Her typical seizures begin with a rising epigastric sensation that progresses to loss of awareness or may begin without warning and consist of an abrupt behavioral arrest and loss of contact. Postictally, she is confused, but denied any interference with speech or comprehension. MRI showed right hippocampal sclerosis, and her scalp EEG demonstrated right temporal ictal and interictal epileptiform abnormalities.

This patient's neuropsychological testing results are shown in Table 1. Her intellectual abilities and functioning in most cognitive domains were within normal limits, though there was a significant discrepancy between visuospatial perceptual/constructional skills (average) and verbal abilities (borderline), with the latter representing a clear area of weakness. Her performance on memory tests revealed deficits in *both* verbal and visual domains without any significant discrepancies between the two modalities. The dichotic listening task showed a strong left ear advantage (LEA). fMRI to confirm language dominance was recommended because LEA is generally an unusual finding. Also, in this case operating on the dominant hemisphere would increase the chances of anterograde amnesia after surgery.

Language maps for Case 3 (Figure 4c) showed reliable task‐related activation in the *left* middle temporal gyrus/sulcus and somewhat less robust activation in the *left* inferior frontal gyrus. Additional activation was noted in the motor and SMA regions on the left, which is a typical finding for this paradigm. In the right hemisphere, there were very small areas of task‐related activation, mainly in the right middle temporal sulcus.

fMRI findings of predominantly left hemisphere language representation were discrepant with LEA on dichotic listening and with a weakness in verbal abilities in presence of right temporal lobe epilepsy. Once again, although behavioral data is arguably more vulnerable to variation and error, it cannot be discounted in this case since it was consistent with EEG and MRI findings. Though it is possible that greater right hemisphere involvement in language processing may have been observed with a different panel of tasks, it would be unlikely given the comprehensive nature of our paradigm. As such, it appeared that all methods were technically adequate and none could be discounted in favor of others. Since no conclusions regarding language dominance could be reached on the basis of noninvasive testing, and given deficits in both verbal and visual memory (i.e., a risk of amnesia following temporal lobe surgery), an eSAM test was recommended.

This individual completed an eSAM test at the Montreal Neurological Institute several months following her fMRI. Language testing showed significant speech disturbance following a left‐sided etomidate injection and more subtle, but significant, speech errors following the right injection. These findings were thought to represent bi‐lateral speech representation, with a greater involvement of the left hemisphere. This is in keeping with previous reports that fMRI may be less useful and more challenging to interpret in individuals with bilateral speech representation (Bauer et al., 2014). Memory testing was failed after both the left and the right injections, but her memory score was much worse following the left injection. Together with speech findings, memory results suggested low risk of significant memory decline or amnesia after a right temporal lobe resection.

This patient was seen for a follow‐up neuropsychological assessment 1 year following her right temporal lobe resection. She reportedly experienced one generalized tonic clonic seizure several months following the resection, but otherwise remained seizure‐free. Her neuropsychological profile remained stable with the exception of declines in nonverbal learning and memory. Although declines were noted on some tasks of verbal learning, her performance improved significantly when she was provided with opportunity for depth of processing at encoding (e.g., encouragement to think about the stimuli in greater detail during learning of words). Such improvement was not observed with respect to nonverbal material, like faces. As such, it is most likely that this change in effortful verbal learning was secondary to extremely low mood and anxiety, which developed for the first time following surgery and persisted at the time of the reassessment.

### 2.4 Caveats

All of the examples above were in reference to native English‐speakers, and patients without large lesions in the “eloquent” zones. Determining language dominance in individuals whose first language is not English, or those with structural abnormalities in peri‐Sylvian areas is more challenging. A detailed example of how we have handled dichotic/fMRI decision‐making with a bilingual patient with limited English proficiency is described in O'Grady, Omisade, and Sadler (2016). In rare cases when we have seen individuals with large structural lesions affecting traditional language areas, we have tended not to rely on fMRI findings due to higher probability of abnormal neurovascular coupling and false negative findings. In those cases, we have recommended stimulation mapping for language in/around the regions targeted for resection. In general, approaches in those cases must be more individually‐tailored and will depend on a great number of individual variables that are beyond the scope of this article.

## 3 DISCUSSION

The situations presented above highlight the challenges of determining language laterality in individual patients for the purposes of surgical planning in epilepsy. fMRI has been promoted by some researchers as highly reliable and valid method that could replace both dichotic listening and “the gold standard” for determining language dominance, which is intracarotid testing like Wada or eSAM. However, cases of disagreement between fMRI and other clinical indicators of language dominance, or situations where fMRI is not easily interpretable or helpful, are frequent (Benjamin et al., 2018). This is to be expected, since most individuals are referred for fMRI are subject to selection bias: most demonstrate a disagreement among other indicators of laterality and are already considered to be “complex” with regard to determining language dominance. Disagreement among other clinical indices of language dominance are also themselves quite common in TLE (Gargaro et al., 2013).

In this article, we have presented three scenarios where fMRI was discrepant with other clinical indices of dominance or was otherwise difficult to interpret with regard to determining language dominance. We have described the ways in which we have approached these situations clinically on individual bases in our center. We wish to highlight several important points: (a) in situations where neuropsychological findings are uninformative or discrepant with fMRI, we generally tend to place greater weight on fMRI findings, provided that they do show clear lateralization; (b) even strongly‐lateralizing fMRI findings should be carefully interpreted in context of other surrogate markers of language dominance; (c) if bilateral language representation is strongly suspected, fMRI may not be a reliable tool in determining dominance.

When language dominance cannot be predicted with confidence using noninvasive methods, one approach we have recommended is treating the surgical hemisphere as dominant to avoid/limit loss of function. However, this is not always possible or practical, especially if a larger resection is required to alleviate seizures. These are the situations where more invasive procedures, like stimulation language mapping or intracarotid testing, are recommended (Barr & Morrison, 2015). Ultimately, methods like IAP or eSAM remain the gold standard for determining lateralization of function in patients with epilepsy. It is still the most reliable way of determining hemispheric dominance and functional language and memory reserve in complex cases where there is a lack of agreement among clinical indicators of laterality (Arora et al., 2009). Furthermore, some recommendations and resulting guidelines for the use of fMRI in presurgical planning for epilepsy suggest there is weak evidence for the Wada/IAP test to be completely replaced with language fMRI and that choice of fMRI or IAP requires careful clinical decision making (Szaflarski et al., 2017). While no longer recent, the sentiment of the ILAE Commission report describing fMRI as a rapidly evolving clinical tool remains relevant in practice today (Kuzniecky, McLachlan, Sadzot, & Theodore, 1998). In 50% of European centers, for example, fMRI is used to confirm language dominance for patients where atypical dominance is expected and therefore presumably not as a replacement for neuropsychological and other tests for language dominance, including IAP (Vogt et al., 2017).

In order to increase the clinical reliability and usefulness of fMRI, further research is required, particularly with regards to the factors that contribute to inaccuracies in fMRI. We do not always understand what factors have played a role in individual cases, what other patient‐specific variables (if any) may have impacted the findings, and also how to adjust for these variables so as to yield the most accurate findings in an individual patient. This will involve creating standard fMRI protocols, validating them and collecting normative data from healthy populations and a range of clinical populations. Currently, standard paradigms are lacking (Benjamin et al., 2018).

## CONFLICT OF INTEREST

The authors have no conflict of interest to declare.

## DATA AVAILABILITY STATEMENT

The data that support the findings of this study are available on request to qualified clinicians or researchers from the corresponding author. The data are not publicly available due to privacy or ethical restrictions.
